# A Predictive Spatial Model to Quantify the Risk of Air-Travel-Associated Dengue Importation into the United States and Europe

**DOI:** 10.1155/2012/103679

**Published:** 2012-03-14

**Authors:** Lauren M. Gardner, David Fajardo, S. Travis Waller, Ophelia Wang, Sahotra Sarkar

**Affiliations:** ^1^School of Civil and Environmental Engineering, The University of New South Wales, Sydney, NSW 2052, Australia; ^2^Department of Geography and the Environment, The University of Texas at Austin, Austin, TX 78712, USA; ^3^Patterson Laboratories, Section of Integrative Biology, The University of Texas at Austin, Austin, TX 78712, USA

## Abstract

The number of travel-acquired dengue infections has been on a constant rise in the United States and Europe over the past decade. An increased volume of international passenger air traffic originating from regions with endemic dengue contributes to the increasing number of dengue cases. This paper reports results from a network-based regression model which uses international passenger travel volumes, travel distances, predictive species distribution models (for the vector species), and infection data to quantify the relative risk of importing travel-acquired dengue infections into the US and Europe from dengue-endemic regions. Given the necessary data, this model can be used to identify optimal locations (origin cities, destination airports, etc.) for dengue surveillance. The model can be extended to other geographical regions and vector-borne diseases, as well as other network-based processes.

## 1. Introduction

Dengue is the most common mosquito-borne viral diseases in the world [1]. Although it is not currently endemic to either Europe or the continental United States, except along the Texas-México border and possibly Florida, an increase in dengue occurrence in many of the endemic regions worldwide, in conjunction with a significant rise in the volume of international air travel, has resulted in a greater likelihood of imported dengue infections among travelers returning to the United States and Europe from dengue-endemic regions [2]. It has also increased the potential for transport and establishment of the mosquito vector species in those regions of Europe and the U.S. in which suitable habitat is available.

The causal agent for dengue is a virus that is transmitted from person to person through the bite of infected *Aedes* mosquitoes (mainly *Ae*. *aegypti* and *Ae. albopictus*), with humans serving as the main viral host [1]. The geographic establishment of dengue is thought to be limited purely by the occurrence of its principal vector mosquito species, *Ae. aegypti* and *Ae. albopictus*. Both species have proven to be highly adaptable to human habitation and, as a result, the global spread of the vector has been difficult to contain [1]. Dengue is considered endemic to urban and suburban areas in parts of tropical and subtropical America, part of Australia, South and Southeast Asia, the Pacific, and eastern Africa. In addition, the number of imported cases of dengue in the U.S. and Europe is on the rise and further spread and establishment are anticipated [2, 3].

At present, there is no epidemiological surveillance on a national scale in Europe or at the state level in the U.S. [3]. Limiting the importation and establishment of dengue will require dedicated surveillance measures, ideally based on reliable models of vector presence and virus incidence. This paper presents an extendible preliminary model which prioritizes passenger air travel routes for high likelihood of importing infection into the U.S. and Europe from dengue-endemic regions. Our network-level regression model uses air traffic volumes, travel distances, predictive species distribution models, and infection data, to quantify the relative likelihood of importing infection along air travel routes. More precisely, this paper has two goals.

- Development of a model that allows quantification of the risk of dengue importation through specific air travel routes, thus identifying locations at which surveillance systems can optimally be implemented.
- Prioritization of the type of data collection efforts that must be undertaken to enhance the predictive accuracy of such models.

Given the requisite data, our model can be used as a prediction tool for assessing the risk of importing dengue-infected humans or vectors via air travel based on origin-destination pairs as well as to analyze the effects of changes in passenger travel routes and volumes on spatial patterns of infection spread.

 The model compounds all modes of dengue infection which can be caused by four virus serotypes (DENV-1, DENV-2, DENV-3, and DENV-4), and can range in clinical manifestation from asymptomatic infection to severe systemic disease [1]. Dengue fever (DF) is the more common manifestation of the virus (with an estimated 50 million infections occurring annually world wide), while dengue haemorrhagic fever (DHF) and dengue shock syndrome (DSS) are rarer and much more severe manifestations of the disease. The model presented in this paper does not distinguish between DF, DHF, and DHS cases because the data available do not permit a more fine-tuned analysis.

Although dengue is now rare in the U.S. and Europe, the mosquito vectors are still present. At least one of the two major vector species, *Ae. aegypti *or* Ae. albopictus*, is known to have established populations in many U.S. states [3]. The European Center for Disease Control [4] recently gathered entomological and environmental data to map the current distribution, as well as establishment risk, for *Ae. albopictus* in Europe in the event of its introduction. It concluded that temperate strains of this species already exist and are likely to spread with new populations becoming established in several parts of Europe [4].

Thus, imported cases of dengue via international travel may potentially result in establishment of an autochthonous disease cycle and new regional outbreaks in both the U.S. and Europe. This can occur in at least two ways: (i) locally established mosquito populations become infected from new hosts (infected travelers) and then spread the disease; or (ii) mosquitoes carrying the virus arrive at a new environment suitable for them. This threat was exemplified recently in Key West, Florida, which experienced sizeable local outbreaks of autochthonous dengue transmission in 2009-2010 [5]. There have also been dengue outbreaks in south Texas, along the Texas-Tamaulipas border, but air travel is unlikely to have had a role in these outbreaks [6].

Epidemics of dengue, their seasonality, and oscillations over time, are reflected by the epidemiology of dengue in travelers [2]. Modern transportation bridges the natural barriers previously responsible for containing infected vectors to a specific geographic region. For example, the global movement of troops and cargo ships during World War II facilitated the dissemination of Aedes mosquitoes and resulted in substantial spread of the disease in Southeast Asia [7]. Transportation of used tires has been shown to be responsible for introducing *Ae. albopictus* into the U.S. from Brazil in the 1980s [2].

Various studies have been conducted to identify the highest travel risks. One survey conducted by the European Network on Imported Infectious Disease Surveillance program [8], analyzed 294 patients with DF for epidemiological information and clinical features. They found most infections were imported from Asia [9]. Tatem et al's. [10] estimated the relative risk of the importation and establishment of *Ae. albopictus *by sea and air routes, based on normalized measures of traffic and climatic similarity, and found a strong positive correlation between the historic spread of *Ae. albopictus *(into new regions) and a high volume of shipping (routed from ports where the species was already established). The total volume of travel was determined by the number of ship visits for sea travel and passenger volume for air travel. The climatic similarity was calculated as a distance-based vector.

Tatem et al. [10] approach can be extended through quantitative validation of such models. While their work provided insight into the vector importation and establishment process, model validation remained qualitative. In this paper we extend that approach by complementing qualitative risk analysis with quantitative model calibration using infection data. Moreover, Tatem et al.'s approach addressed the risk of importation and establishment of the vector but not the likelihood of infection directly. Our analysis takes infected individuals into account. Additionally, we incorporate climatic factors using species distribution models which are more robust than statistical correlational analysis as relied upon by Tatem et al. This methodology has become standard in disease ecology and epidemiology [11–13].

## 2. Models and Methods

Our analysis quantifies the relative risk of dengue infected (air travel) passengers entering currently nonendemic regions in the U.S. and Europe at which dengue cases have been recorded. However, it does not include the importation of infected vectors since the influence of that possibility is yet to be established [1, 2, 4]. This section further motivates the problem and introduces a network-based regression model for the risk analysis.

### 2.1. Imported Dengue in the United States and Europe

Nearly all dengue cases reported in the 48 continental U.S. states were acquired elsewhere by travelers or immigrants. From January 1996 to the end of December 2005, 1196 cases of travel-associated dengue were reported in the continental U.S. [14] (most dengue cases in U.S. nationals occur in those inhabitants of noncontinental U.S. territories such as Puerto Rico (with over 5000 cases reported in 2005), the U.S. Virgin Islands, Samoa, and Guam, which are all endemic regions). In 2007, an estimated 17 million passengers traveled between the U.S. mainland and dengue-endemic areas of Asia, the Caribbean, Central and South America, and Oceania [15]. Since 1999 there have been 1117 cases of dengue in European travelers reported to the European Network on Imported Infectious Disease Surveillance [8].

 Further complications arise from the severe underestimation of dengue cases due to underreporting and passive surveillance in both endemic and non-endemic regions. In tropical and subtropical countries where dengue fever is endemic, under-reporting may be due to misdiagnosis, limitations of the standard World Health Organization (WHO) case classification, and lack of laboratory infrastructure and resources, among other factors [16]. In non-endemic regions such as the U.S. and Europe, the actual number of dengue infections is greatly underestimated due to unfamiliarity with the disease. Additionally, 40–80% of all dengue infections are asymptomatic and closely mimic flu symptoms for which they are mistaken. This lack of accurate infection data makes it difficult to assess the actual threat of the disease.

### 2.2. Data

The species distribution models required data on the geographical occurrence of *Ae. aegypti* and *Ae. albopictus* and a suite of predictive environmental variables which will be discussed in Section 2.3.

 The required data for the network model were as follows.

1. Disease data: annual infection reports for dengue-endemic countries, non-endemic European countries and U.S. states.
2. Transportation data: passenger air traffic volumes for all flights originating from endemic regions and destined for Europe or the U.S.
3. Spatial data: the corresponding distances for all travel routes.

Unliess explicitly indicated otherwise, the data used in this model were from 2005, and aggregated to the annual level.

The set of dengue-endemic countries was as identified by the CDC [17]. Country-level infection data for the endemic regions and for European countries were obtained from the regional offices of the World Health Organization [18]. U.S. state level infection data was taken from the CDC [14]. These data sets include the annual number of reported cases for 2005 and 2007, the average of which was used to calibrate the model. Infection data were incorporated in the model in two ways. The number of reported cases at an endemic region was treated as an independent variable in the model, while infection reports for the susceptible node sets (U.S. states and E.U. countries) were used to calibrate the model.

Difficulties were encountered in acquiring the necessary infection data. First, surveillance data for dengue in Africa were sparse. Even though all four dengue virus serotypes have been documented there [19], we were unable to secure country-level infection data for most African countries. Consequently, these endemic countries were ignored in the model. Although model performance is likely to improve if such data could be incorporated, available reports indicated that Africa is responsible for a relatively small fraction of travel-acquired dengue infections [20]; thus these countries appear to be the unlikely to have a significant impact model predictions. We were also unable to gather infection data for certain endemic countries in the western Pacific region which were similarly ignored and presumed not very relevant.

Transportation data were obtained from two sources. The U.S. air traffic data were from the Research and Innovative Technology Administration (RITA), a branch of the U.S. Department of Transportation (U.S. DOT), which tracks all domestic and international flights originating or ending in the U.S. and its surrounding regions [21]. Passenger market data was aggregated by World Area Code (WAC) to determine the total volume of passengers traveling from each endemic country into any U.S. state in 2005. A similar analysis was done using passenger air traffic data from Eurostat [22] to determine the volume of passengers flying into each European Union country from each endemic country. The transportation data used in this paper focus on passenger travel volumes and do not include cargo flights on which vectors could potentially be transported because the latter mode of dengue spread was excluded from this model.

The average distances used in the model were calculated in ArcGIS 9.3. The average distances were computed for each route as the geodesic distance between the geographic centers of each region.

### 2.3. Species Distribution Models

The risk for the establishment of dengue and potential cases of disease in an originally non-endemic area depends fundamentally on the ability of a vector to establish itself in that area. If the vector can establish itself then the disease can become endemic in two ways: (i) if the vector is already established, it can become infected from a person infected with dengue arriving in that area; or (ii) infected vectors can be transported into such an area and establish themselves. For this process, habitat in that area must be ecologically suitable for that vector. A relative measure of the suitability of one area compared to another defines a measure of the relative ecological risk [11–13]. If the ecological risk is low, such an establishment is highly unlikely. If that risk is high, then other factors, such as the (temporally) immediate ambient environmental conditions and the size of the founder population or the availability of hosts, become critical for establishment.

The analysis here was based on habitat suitability for the two principal dengue vector species, *Ae. aegypti* and *Ae. albopictus*. It was assumed that these two species do not interact, that is, the probability of the presence of each is independent of that of the presence of the other. The relative ecological risk for the establishment for each species was estimated using a global species distribution model at a 1 arc-minute resolution [23, 24] based on a maximum entropy algorithm incorporated in the Maxent software package Version 3.3.4, [25]. Maxent was used because it has proven to be predictively superior to other species distribution modeling algorithm in a large variety of studies [24, 26]. As input, Maxent uses species occurrence points (presence-only data) and environmental layers (the explanatory variables). The former were obtained from the Disease Vectors database [27]. The latter consist of four topographic variables (elevation, aspect, slope, and compound topographic index) and a standard set of 19 climatic variables all derived from the WorldClim database [28, 29]. Models were constructed using a variety of subsets of these environmental variables. All computations used default settings [26]. Averages over 100 replicate models were computed. The best model was judged using the Akaike Information Criterion (AIC) for species distribution models. The best model for *Ae. aegypti* is one that used all 23 explanatory variables; that for *Ae. albopictus* is based on elevation, slope, aspect, maximum temperature of warmest month, minimum temperature of coldest month, precipitation of wettest month, and precipitation of driest month. Details of the species distribution models will be published separately in the epidemiological literature.

The output from Maxent consists of relative suitability values between 0 and 1 which, when normalized, can be interpreted as the probabilistic expectation of vector presence of a species in a cell. The probabilistic expectation of at least one of the vector species being present in a cell was calculated as the complement of the probability that neither is present, assuming statistical independence. Because the infection and travel data used in this work are at the state level for the U.S. and the country level for Europe, the expectations are aggregated to the same level by averaging them over all the cells in the relevant geographical units. These expectations define the relative ecological risk for dengue in each cell.

### 2.4. Mathematical Network Model

The network model predicts the expected number of dengue cases in each non-endemic region that can be attributed to a particular endemic region connected to it by travel. Two previous mathematical models quantifying risk estimates for acquiring arboviral infection are by Massad and Wilder-Smith [30] and Codeço et. al. [31]. Massad and Wilder-Smith's model was intended to evaluate the risk of infection at a specific site as a function of human population size, the number of infected mosquitoes, and estimated parameters for the biting rate and the probability that an infectious mosquito will infect a susceptible human. The model did not incorporate travel patterns or species distribution data; moreover, model predictions were not quantitatively validated using infection data. Codeço et. al. assessed the risk of yellow fever (YF) emergence in the city of Rio de Janeiro, Brazil, by estimating the probability of infected individuals arriving from YF-endemic areas via air and bus travel, and the probability of infective individuals triggering an epidemic (by using a stochastic transmission model). While this model accounted for travel patterns and local transmission probabilities, the model predictions were again not quantitatively validated.

Our model has similarities to a feedforward artificial neural network (ANN). Feed-forward ANNs have been used to model learning input-output systems, and can be calibrated through a “back-propagation” algorithm that minimizes a cost function representing output error [32]. The approach taken in this paper differs from traditional implementations of ANNs insofar as, not only is a response function calibrated, but the function itself must be chosen to suit the process.

#### 2.4.1. Network Structure

In the proposed network structure, geographic areas were represented as nodes, belonging to either the set *G* of endemic nodes, or one of the sets *N*_*U*_ or *N*_*E*_ of susceptible nodes in the United States and Europe, respectively. The links in the network represent directed air travel connections between geographic areas (originating from *G*), while the measure *P*_*ji*_ represents the number of predicted infections at a susceptible node *i* attributed to an endemic node *j*.

**Figure 1:**
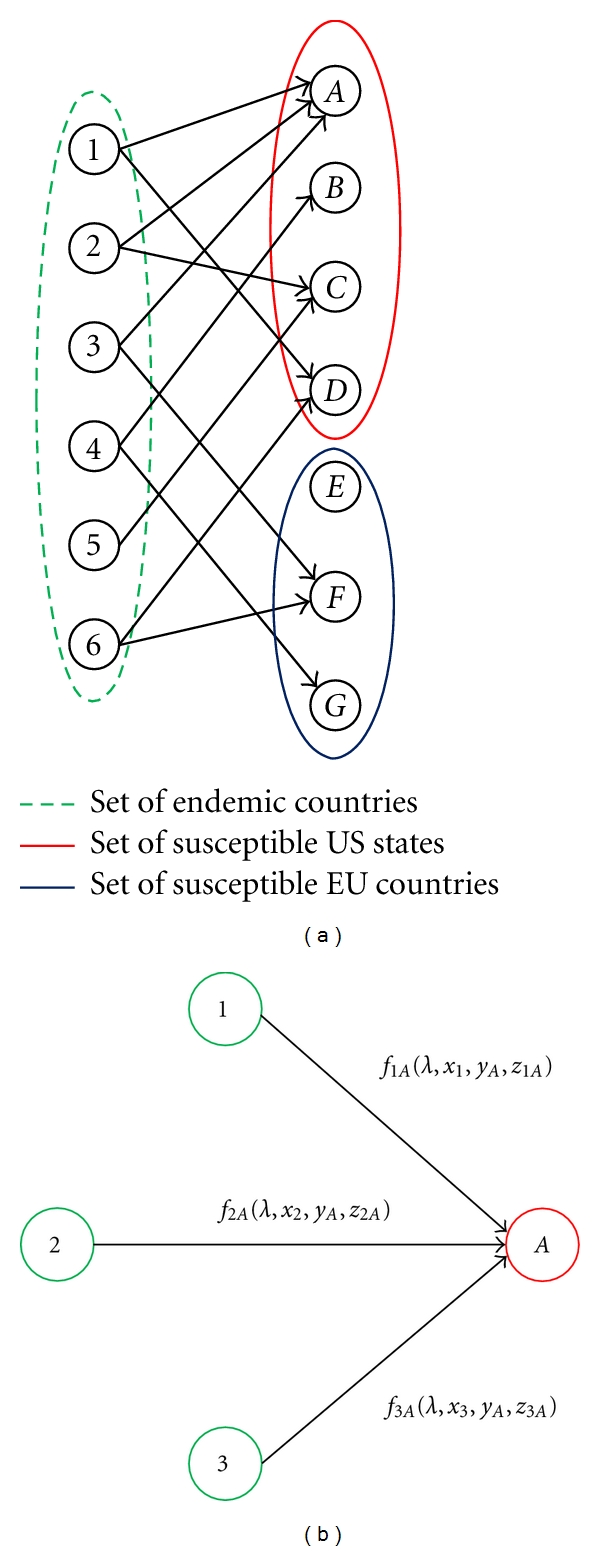
(a) Bipartite network connecting endemic regions to susceptible regions: the susceptible U.S. and Europe nodes represent mutually exclusive sets; (b) link-based functions: these predict the number of infections at susceptible node A, attributed to each adjacent endemic region (1, 2, and 3).

This directed bipartite network structure connected endemic countries to susceptible regions (U.S. states and E.U. countries). Initially a single model was developed which included all susceptible regions as a single set of destination nodes, *N*. However the significantly higher number of reported infections in Europe relative to the U.S. resulted in extremely poor predictions. The limited performance was likely a result of unobserved variables which differentiate the risk of importing infection into Europe versus the U.S, such as border control procedures, quality of healthcare and quality of disease surveillance. These variables were difficult to quantify directly, as we found through empirical testing, and were best accounted for by modeling the U.S. and Europe separately.  Figure 1(a) provides an example of a bipartite network structure representative of the network structure modeled in this paper. The network modeled in this work was limited to the regions with available infection data. The resulting network included 56 endemic nodes, 42 total susceptible nodes (30 U.S. states and 12 European countries), and 664 links. The reason the network was not fully connected is because passenger travel did not occur between all pairs of nodes.

Figure 1(b) is a four-node extraction from the example network to illustrate the generalized link-based functional form used in our model. The function *f*_*ji*_(*λ*,*x*_*j*_, *y*_*i*_, *z*_*ji*_) represents the number of cases observed at *i* for which *j* is responsible, where *λ* represents a vector of calibrated parameters, *x*_*j*_ represents the characteristics of origin *j*, *y*_*i*_ represents the characteristics of destination *i*, and *z*_*ji*_ represents the vector of parameters specific to directed link (*j*, *i*). The total predicted number of infections at *i* is *P*_*i*_ = ∑_∀*j*∈*A*(*i*)_ *f*_*ji*_(*λ*,*x*_*j*_, *y*_*i*_, *z*_*ji*_), where *A*(*i*) represents the set of endemic nodes adjacent to *i*. 

The most critical issue was determining the functional form of *f*_*ji*_(*λ*,*x*_*j*_, *y*_*i*_, *z*_*ji*_). Two complications arose: first, the process that *f*_*ji*_(*λ*,*x*_*j*_, *y*_*i*_, *z*_*ji*_)  attempts to model was too complex to determine a functional form *a priori*, that is, the relative impact different variables will have is not clear ahead of time. Second, directional infection data (i.e., the source of infection for travel acquired dengue cases) was not available. Consequently, specifying the functional form of *f*_*ji*_(*λ*,*x*_*j*_, *y*_*i*_, *z*_*ji*_) was not feasible. Because the objective was to identify a link-based functional form that best replicated the number of reported cases at each susceptible region, a variety of functional forms were examined to identify the one with optimal performance.

#### 2.4.2. Problem Formulation

The notation used in the formal problem formulation is shown in Table 1.

The purpose of this analysis was to examine a variety of families of functions, further explore the most suitable member of each family, and examine the results from a qualitative perspective. The objective was to find the parameter vector *λ* for a given *f*_*ji*_(*λ*,*x*_*j*_, *y*_*i*_, *z*_*ji*_) such that the difference between  *I*_*i*_, the observed number of infections at susceptible node *i*, and *P*_*i*_, the predicted number of infections at *i*, was as small as possible. To ensure this, we formulated a nonlinear convex program to find the unknown parameter vector *λ* which minimized the sum of the squared difference between observed and predicted infection values over all susceptible nodes in the set. The problem formulation is as follows:

(1) $$\begin{array}{cc} & \underset{\lambda }{\min\;}\underset{\forall i\in N}{\sum }{\left(I_{i}-P_{i}\right)}^{2}\\ & \text{s}.\text{t}.\;P_{ji}=f_{ji}\left(\lambda {,x}_{j},y_{i},z_{ji}\right)\;\forall i\in N\;\forall j\in G\\ & \;P_{i}=\underset{\forall j\in A\left(i\right)}{\sum }P_{ji}\;\forall i\in N. \end{array}$$

The characteristics of the resulting linear program depend on the role of the parameter vector *λ* in the function, *f*_*ji*_(*λ*,*x*_*j*_, *y*_*i*_, *z*_*ji*_). If the function is linear in respect to *λ*, the resulting program can be solved analytically for the optimal decision parameters through a system of linear equations. In other cases, however, the resulting function may be non-convex, and as such solvable only through simulation.

#### 2.4.3. Functional Forms

Depending on the functional form of *f*_*ji*_(*λ*,*x*_*j*_, *y*_*i*_, *z*_*ji*_), namely, the behavior of *f*_*ji*_(*λ*,*x*_*j*_, *y*_*i*_, *z*_*ji*_)  with respect to *λ*, the tractability of the resulting mathematical program will vary. In developing a sensible link function, we considered several factors such as the highly nonlinear response of the explanatory variable with respect to the dependent variables considered and concerns about overfitting the data. Various functional forms were examined and compared, and the best performing function was found to have the following form:

(2) $$P_{ji}=\beta +\alpha *\;\frac{{V^{\prime }}_{ji}*S_{j}*S_{i}*\sqrt{{I^{\prime }}_{j}}\;}{\sqrt{{\;D^{\prime }}_{ji}}\;}\;\forall i\in N,\;\forall j\in G.$$

The motivation for the final functional form, *P*_*ji*_  defined above, came from the Gravity Model for Trip Distribution. The function is the sum of two terms: the first term on the RHS is equivalent to the constant term in a standard regression model; while the second term bears a strong resemblance to the Gravity Model used for trip distribution. In the Gravity Model the fraction of trips attracted to zone *j* from zone *i* is proportional to the population of both zones, and inversely proportional to some measure of generalized cost of travel between them. Similarly, in the second term of the RHS of the equation above, the numerator accounts for the travel volume, the relative ecological risks of the origin and destination (from the species distribution models), and the number of cases reported at the source, while the distance is included in the denominator.

The square root of *I*_*j*_′ represents the concave relationship between the predicted number of infections at a susceptible location and the number of reported cases at an endemic source. For the denominator, the lowest value for the sum of squared errors was obtained by taking the square root of the distance. While proximity to endemic countries showed a positive correlation to the reported cases, the differential effect of distance was higher for areas closer to endemic regions. The concavity of the term can be attributed to the relationship between travel time and distance, which is certainly not linear. In order to normalize the data, the values for travel volume, distance, and number of reported cases at endemic regions were rescaled by the maximum value across all observations for their respective category.

#### 2.4.4. Model Parameter Estimation

By rewriting the original mathematical program in terms of the node based variables *P*_*i*_, it is evident that it holds the same structure as a multiple linear regression. The model was solved using the Ordinary least squares estimation procedure:

(3) $$\begin{array}{c} \underset{\lambda }{\min}\;\underset{\forall i\in N}{\sum }{\left(I_{i}-P_{i}\right)}^{2}, \end{array}$$

where

(4) $$\begin{array}{c} P_{i}=\beta *\xi \left(i\right)+\alpha *\varphi \left(i\right)\;\forall i\in N,\\ \varphi \left(i\right)=\underset{\forall j\in A\left(i\right)}{\sum }\frac{{V^{\prime }}_{ji}*S_{j}*S_{i}*\sqrt{{I^{\prime }}_{j}}\;}{\sqrt{{\;D^{\prime }}_{ji}}\;}\\ \xi \left(i\right)=\left|A\left(i\right)\right|. \end{array}$$

In order to estimate the values of *α* and *β*, we solved the system of equations that resulted from the first-order optimality conditions of the convex program shown above. The system of equations reduced to:

(5) $$\begin{array}{c} \underset{i}{\sum }I_{i}\xi \left(i\right)-\alpha \underset{i}{\sum }\xi \left(i\right)\phi \left(i\right)-\beta \underset{i}{\sum }\xi \left(i\right)\xi \left(i\right)=0,\\ \underset{i}{\sum }I_{i}\phi \left(i\right)-\alpha \underset{i}{\sum }\phi \left(i\right)\phi \left(i\right)-\beta \underset{i}{\sum }\phi \left(i\right)\xi \left(i\right)=0. \end{array}$$

Solving the system of equations yielded as estimates for *α* and *β*:

(6) $$\begin{array}{c} \alpha =\frac{\sum_{i}I_{i}\xi \left(i\right)\sum_{i}\xi \left(i\right)\phi \left(i\right)-\sum_{i}I_{i}\phi \left(i\right)\sum_{i}\xi \left(i\right)\xi \left(i\right)}{\sum_{i}\xi \left(i\right)\phi \left(i\right)\sum_{i}\xi \left(i\right)\phi \left(i\right)-\sum_{i}\xi \left(i\right)\xi \left(i\right)\sum_{i}\phi \left(i\right)\phi \left(i\right)}\\ \beta =\frac{\alpha \sum_{i}\phi \left(i\right)\phi \left(i\right)-\sum_{i}I_{i}\phi \left(i\right)}{\sum_{i}\xi \left(i\right)\phi \left(i\right)}. \end{array}$$

## 3. Results and Discussion

The main objective of the model was to quantify the relative risk of various international travel routes. This was accomplished by first predicting the number of dengue cases specific to each travel route, and then calibrating the network model at a regional level using infection data. Therefore, there are two sets of results presented.  Section 3.1 includes the total number of dengue cases predicted for each susceptible region based on the calibrated model output, and Section 3.2 includes the corresponding relative risk of each travel route, ranked based on their likelihood of transporting infected passengers.

The results included in this section are representative of filtered data. The filtering process was applied to the susceptible node set to remove outliers. The outliers were classified differently for the European and U.S. node sets. In the European data set any region with less than 5 cases was considered an outlier, while only states with one reported case were considered outliers in the U.S. node set. A lower threshold was implemented for the U.S. as there were fewer reported cases on average. The procedure resulted in five nodes being removed from *N*_*E*_ and 12 nodes being removed from *N*_*U*_. After the filtering process there were 18 U.S. states and seven European countries included in the model.

### 3.1. Susceptible Node-Based Predictions

The model was able to predict closely the number of reported cases for the European countries, though it struggled to predict the number of reported cases for the U.S. states accurately. The results for the node-based predictions, *P*_*i*_, are shown in Table 2(a) for European Countries and Table 2(b) for U.S. states.

The functional form introduced in Section 2.4.3 was used in both models, while the resulting regression parameters, *α* and *β* had different estimates. For Europe the optimal *α* and *β* were 271.52 and 5.08, respectively; for the U.S. 5.54 and 0.595. The combination of the low constant (*β*), high *α* value, and good fit of the European model signifies that the majority of variability in the data was accounted for by the independent variables included in the model. This was not the case with the U.S. model. On average, the European model predictions diverged from the reported cases by 24, where 112 actual cases were observed on average per node. The U.S. model predictions diverged from the reported cases by an average of 6.2, where an average of 10.4 cases were reported per node.

Several factors contributed to complicating the task of identifying a function to perfectly fit the case data. Firstly, the limited size of the susceptible node set made it difficult for the model to differentiate between variability and noise. Secondly, the amount of noise in the data due unknown factors such as variations in regional surveillance efforts could not be accounted for. Thirdly, prevention measures being implemented were not only difficult to determine, but also difficult to quantify. All these uncertainties restricted the model's ability to estimate parameters that resulted in good predictive properties at the node level. However, our results show that, though the fit at the node level could be improved upon, the route-level risk measures do show promising results, and as such, provide some insight into the role the independent variables play.

### 3.2. Endemic-Susceptible Route-Based Risk

Although the node-based predictions can be validated based on the reported infection data, the resulting route-based predictions were not directly-verifiable due to the unavailability of route-based infection data. The best measures of validation were (i) to find route-based predictions that correspond to known regional infection data when summed across all incoming routes, and (ii) to compare the results with previous travel-based patient surveys conducted to determine the most likely place of origin for illness.

Table 3 identifies the 20 international travel routes with the highest probability of carrying dengue-infected passengers into (a) Europe and (b) the US, and their corresponding relative risk, as produced by the model. The initial ranking was determined based on the predicted number of infected passengers traveling on each route. The predicted number of infected passengers was then normalized to the highest ranked route. Although the results shown are specific to the filtered node sets, similar results were obtained for the full node sets, for both Europe and the U.S. In the model Burma, Cambodia, Laos, and Thailand were aggregated to a single “South East Asia” endemic region.

**Figure 2:**
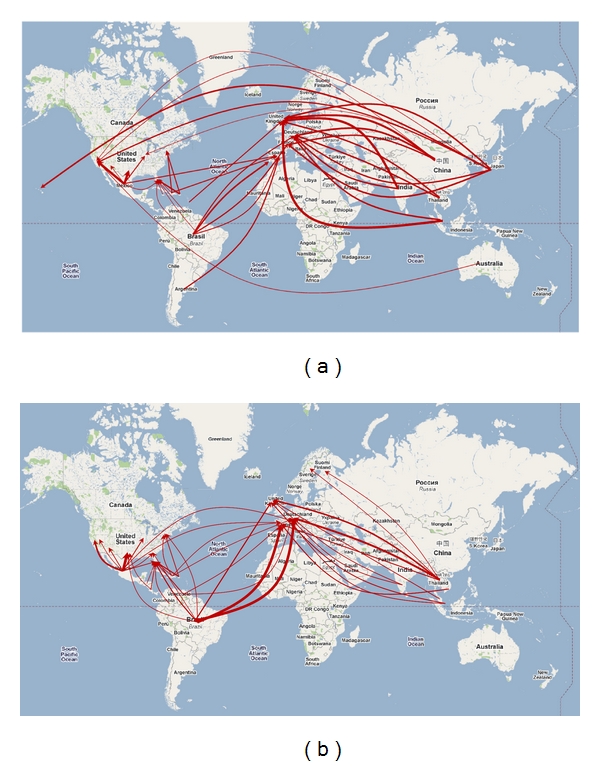
(a) The 20 highest traveled routes entering the U.S. and E.U. There are 40 total links; the line thickness is proportional to the travel volume. (b) The top 20 travel routes with highest relative risk of carrying Dengue infected passengers into U.S. and E.U. The line thickness is proportional to the relative risk of the route.

Figure 2 compares the highest traveled international routes with the highest risk international travel routes for carrying infected passengers (as predicted by the model). The links included in Figure 2(a) are representative of the twenty highest traveled routes entering the U.S. and the twenty highest traveled routes entering the E.U.; the line thickness is proportional to travel volume.  Figure 2(b) provides a visual illustration of the model output, specifically the 20 international travel routes with the highest probability of carrying dengue infected passengers into Europe and the U.S. (the set of links listed in Table 3); the line thickness is proportional to the relative risk of the route. The difference between these two mappings illustrates the significance of the regional level input variables (vector suitability and infection data) included the model.

As stated previously, one way to verify the predicted route-based risk was by comparing the results with previous patient surveys conducted to identify the source of infections. A previous study found of the travel acquired dengue cases in Europe between 1999 and 2002 [33]:

- 219 (45%) originated in South-East Asia, represented in the model as 3 of the top 6 highest risk routes,
- 91 cases (19%) originated in South and Central America, represented in the model as 3 of the top 10 highest risk routes,
- 77 cases (16%) originated in the Indian subcontinent, represented in the model as 2 of the top 15 highest risk routes,
- 56 cases (12%) originated in the Caribbean, represented in the model as 2 of the top 20 highest risk routes.

The model predicted Brazil-Germany and Brazil-France as the two highest risk routes into Europe (with nearly equivalent relative risk). This is expected, as Brazil reported the highest number of dengue cases in the world per year, almost 3-times those of second place Indonesia, and the volume of traffic on the Brazil-France and Brazil-Germany routes were two of the top 40 in the world. Indonesia, reported a very high number of infections, but reported a very low level of air travel on any given route destined for Europe. Using similar logic, Southeast Asia reported a number of infections on par with Indonesia, though the travel volume from Southeast Asia into Germany and the United Kingdom ranked among the world's top 25 travel routes; suggesting intuitively that travel volume is a dominant factor in assessing infection risk.

For the U.S. the model predicted the majority of U.S. infections were attributed to Central and South American countries, likely a result of the close proximity, high traffic, and high level of infection. More specifically, 19 of the top 20 highest risk routes into the U.S. (Nevada, ranked 20th not included) were destined for states which account for a very high fraction of incoming flights in the US; accounting for 6 of the top 15 busiest American Airports by boardings [34].

As a destination, Florida accounted for 5 of the top 10 risk routes, which is supported by historical occurrence of the disease, exemplified in the 2009-2010 local outbreaks. Though it is possible that dengue was already present in the locality (Key West), and previously undetected, the results of this model suggest dengue was likely introduced via international travelers into a locality with environmental and social conditions ripe for transmission [5]; in the model Puerto Rico-Florida ranked as the third highest risk route. The travel volume on this route was among the top ten in the world, while the proximity and climate similarity were likely additional contributors to the infection risk.

Mexico-Texas and Mexico-California ranked as the two highest risk routes, and were also the top two traveled routes (by passenger volume) in the world [34]. The highest risk travel route predicted was from Mexico to Texas; with nearly twice the risk of Mexico-California. The high number of infections reported in Mexico, its proximity to Texas, and the high volume of travel between the two intuitively suggests this to be a high-risk pairing, which is supported by the model.

## 4. Conclusions

Dengue currently presents a serious risk to many parts of the U.S. and Europe where suitable environmental conditions for vector species' occurrence and establishment provide the potential for local outbreaks, were the virus to be introduced. The background to this analysis was the increasing number of dengue cases in the U.S. and Europe, coinciding with an increase in both the prevalence of dengue worldwide and increased volume of international passenger air traffic originating from dengue-endemic regions since the 1990s.

The model presented here was developed to explore the relationship between reported dengue infections and air travel. It used a network-based regression to quantify the relative risk from international air travel routes carrying infected passengers from endemic regions to non-endemic ones in the U.S. and Europe. Besides international passenger travel volumes, the model incorporated predictive species distribution models for the principal vector mosquito species. The model also incorporated travel distances and infection data. The following inferences follow from the model results.

- The highest-risk travel routes suggest that the proximity to endemic regions is a dominant factor. Most high-risk routes into Europe originate in Asia (with the exception of Brazil and Mexico), while all top 20 routes into the U.S. originate in South and Central America.
- Travel from dengue-endemic countries presents significant risk to Florida. Additionally, the high volume of domestic visitors to Florida in conjunction with established *Ae. aegypti* and *Ae. albopictus* populations, enhances Florida's potential role as a fulcrum spreading dengue to other parts of the continental U.S. The recent reemergence of dengue in Florida suggests that strong vector surveillance and control infrastructure is critically needed for identification and control of outbreaks of dengue.
- The high risk predicted for Mexico-Texas travel is further heightened by the risk of overland transmission (such as that from Tamaulipas into the Brownsville area [6]). Therefore surveillance along the Texas-Tamaulipas border should be complemented with surveillance at regions with airports connected to Mexico by regular or chartered flights.
- For many countries of Europe and U.S. states, if dengue gets introduced, the establishment of an autochthonous disease cycle is likely because many of these areas contain suitable habitats for *Ae. albopictus* and some contain established populations.
- Some of the “source” areas indicate that dengue has yet to be brought under control in places where malaria has. This means that dengue may well replace malaria as the paradigmatic airport disease.

The results provided in this paper were obtained using existing (historical) data from the (recent) past and do not represent fully reliable predictions for relative risks in the future. However, the model introduced in this paper can be calibrated using epidemiological data from any time period. The calibrated model can be used as a predictive tool for quantifying route-based risk in the future provided that the necessary data are available, including real-time travel patterns, environmental conditions, and infection data. Moreover, the results in this paper are aggregated at the annual and regional (country or state) level due to the limitations of available data. Infection data proved to be the most difficult to gather because infection reports for many regions in the world are not available even at the annual level. Appropriate data will enable the extension of the model to allow analysis at finer spatial and temporal resolutions: the model can be regionally disaggregated to the city level, or disaggregated by month to account for seasonality. Moreover, this model can be deployed in other geographical regions, used for other vector-borne diseases, and modified to analyze other network-based processes. Finally, this model can potentially be extended to include other modes of transportation, such as freight and shipping networks.

The development of such models is an integral step in improving local and regional surveillance efforts. The quantitative results produced by the model can lead to more specific surveillance recommendations than the CDC is currently able to make such as identifying (i) specific routes on which to implement surveillance and control strategies and (ii) optimal locations (origin cities, destination airports, etc.) for passenger surveillance efforts. As there is currently no vaccine for dengue; surveillance and intervention, along with vector control, are the only relevant options to prevent further geographic spread of the disease. The limitations of this analysis highlight the need for improving the quality of readily accessible disease data so as to enhance the prediction and control of epidemic episodes of vector-borne diseases in susceptible countries.

## Figures and Tables

**Table 1:** Problem Notation.

*N*_*U*_	Subset of susceptible nodes in the United States
*N*_*E*_	Subset of susceptible nodes in Europe
*N*	Complete set of susceptible nodes (*N*_*U*_ ⋃ *N*_*E*_)
*G*	Set of nodes in the endemic region
*I*_*i*_	Number of reported infections at node *i*
*P*_*i*_	Total number of predicted infections at node *i*
*P*_*ji*_	Number of predicted infections at node *i* attributed to node *j*
*λ*	Vector of parameter to be optimized
*x*_*j*_	Vector of characteristics of infecting node *j*
*y*_*i*_	Vector of characteristics of susceptible node *i*
*z*_*ji*_	Vector if parameters specific to link (*j*, *i*)
*V*_*ji*_′	Normalized passenger air travel volume between nodes *j* and *i*, ranging from 0 to 1
*S*_*i*_	Climate suitability of node *i*, ranging from 0 to 1
*I*_*i*_′	Normalized reported infections at node *i*
*D*_*ji*_′	Normalized distance between nodes *j* and *i*, ranging from 0 to 1
*A*(*i*)	Set of endemic nodes adjacent to susceptible node *i*
*α*, *b*	Parameters to be optimize

**Table:** (a) Infections for susceptible European countries.

E.U. country	Actual reported infections	Model reported infections
Belgium	25	31
Czech Republic	9	31
Finland	12	40
France	300	247
Germany	204	231
Sweden	61	55
United Kingdom	170	196

Total	781	831

**Table:** (b) Infections for susceptible U.S. states.

U.S. State	Actual Reported Infections	Model Reported Infections
Hawaii	11	6
Massachusetts	14	12
New York	55	22
Pennsylvania	3	11
Florida	22	24
Georgia	7	16
North Carolina	5	9
Virginia	5	6
Illinois	3	14
Ohio	4	6
Wisconsin	2	4
Minnesota	11	6
Texas	24	20
Arizona	5	4
Nevada	2	7
California	4	22
Oregon	4	4
Washington	6	5

Total	187	196

**Table:** (a) Route-based relative risk european countries.

Rank	From	To	Relative Risk
1	Brazil	Germany	1.00
2	Brazil	France	0.99
3	South East Asia	Germany	0.71
4	South East Asia	United Kingdom	0.52
5	Brazil	United Kingdom	0.35
6	South East Asia	France	0.29
7	Vietnam	France	0.29
8	Singapore	United Kingdom	0.27
9	Singapore	Germany	0.19
10	India	Germany	0.19
11	Malaysia	United Kingdom	0.19
12	India	United Kingdom	0.17
13	Dominican Republic	Germany	0.16
14	Venezuela	Germany	0.16
15	Dominican Republic	France	0.16
16	Mexico	France	0.16
17	Mexico	Germany	0.15
18	Venezuela	France	0.15
19	South East Asia	Finland	0.14
20	South East Asia	Sweden	0.13

**Table:** (b) Route-based relative risk for U.S. states.

Rank	From	To	Relative risk
1	Mexico	Texas	1.00
2	Mexico	California	0.56
3	Puerto Rico	Florida	0.34
4	Brazil	Florida	0.33
5	Venezuela	Florida	0.24
6	Mexico	Illinois	0.23
7	Puerto Rico	New York	0.21
8	Costa Rica	Florida	0.19
9	Mexico	Florida	0.19
10	Mexico	Arizona	0.19
11	Dominican Republic	New York	0.17
12	Colombia	Florida	0.16
13	Brazil	New York	0.15
14	Mexico	Georgia	0.15
15	Dominican Republic	Florida	0.15
16	Brazil	Texas	0.14
17	Brazil	Georgia	0.12
18	Honduras	Florida	0.12
19	Costa Rica	Texas	0.12
20	Mexico	Nevada	0.11
